# Mitochondrial Targeting by Elamipretide Improves Myocardial Bioenergetics Without Translating into Functional Benefits in HFpEF

**DOI:** 10.3390/ijms27021060

**Published:** 2026-01-21

**Authors:** Antje Schauer, Daniela Jahn, Beatrice Vahle, Peggy Barthel, Anita Männel, Gunar Fabig, Axel Linke, Volker Adams, Antje Augstein

**Affiliations:** 1 Laboratory of Molecular and Experimental Cardiology, Department of Internal Medicine and Cardiology, Heart Center Dresden, Faculty of Medicine Carl Gustav Carus, Technische Universität Dresden, Fiedlerstraße 42, 01307 Dresden, Germany; daniela.jahn1@tu-dresden.de (D.J.); beatrice.vahle@tu-dresden.de (B.V.); peggy.barthel@tu-dresden.de (P.B.); anita.maennel@tu-dresden.de (A.M.); axel.linke@tu-dresden.de (A.L.); volker.adams@tu-dresden.de (V.A.); antje.augstein@tu-dresden.de (A.A.); 2 Experimental Center, Faculty of Medicine, Technische Universität Dresden, 01307 Dresden, Germany; gunar.fabig@tu-dresden.de

**Keywords:** HFpEF, Elamipretide, mitochondria, myocardium, vasculature, ZSF1 rat

## Abstract

Mitochondrial dysfunction contributes to impaired myocardial energetics and performance in heart failure with preserved ejection fraction (HFpEF). Elamipretide (Ela) enhances mitochondrial bioenergetics in preclinical models, yet its relevance in HFpEF remains unclear. This study examined the effects of Ela on cardiac mitochondrial function, structure, and cardiovascular performance in a rodent HFpEF model. Female obese ZSF1 rats received vehicle or Ela for 12 weeks, with age-matched lean rats as controls. Cardiac function and hemodynamics were assessed by echocardiography and pressure–volume analysis. Mitochondrial respiration was measured in permeabilized fibers and ultrastructure evaluated by transmission electron microscopy. Molecular and histological analyses included cardiolipin lipidomics and mRNA/protein profiling of hypertrophic, fibrotic, and inflammatory markers. Ela modestly improved complex I and II respiration, whereas mitochondrial ultrastructure, cardiolipin composition, and tafazzin expression were unchanged. Diastolic dysfunction persisted, reflected by unchanged E/é, ventricular stiffness factor β, and titin phosphorylation. Compared to untreated HFpEF, systolic performance showed a mild decline, with small reductions in LV ejection fraction and end-systolic elastance. Accordingly, cardiac remodeling, including hypertrophy, fibrosis, and inflammatory activation, remained unaltered. Vascular stiffness slightly increased, while carotid reactivity and morphology were preserved. In conclusion, despite enhanced mitochondrial respiration following Ela treatment, no functional or structural benefits were observed in experimental HFpEF, suggesting limited therapeutic efficacy once HFpEF is established.

## 1. Introduction

Heart failure with preserved ejection fraction (HFpEF) remains a major clinical challenge, accounting for the majority of all chronic heart failure cases [1]. Characterized by high prevalence, significant morbidity, and limited effective therapeutic options, sodium–glucose cotransporter 2 inhibitors (SGLT2is) and glucagon-like peptide-1 receptor agonists (GLP-1 RAs) have shown some clinical benefit [2,3]. However, overall treatment options for HFpEF remain unsatisfactory. Diastolic dysfunction, a hallmark of HFpEF [4], reflects impaired ventricular relaxation and is increasingly recognized as being linked to mitochondrial dysfunction, which limits ATP supply and compromises diastolic efficiency during the cardiac cycle [5].

At the molecular level, chronic heart failure is accompanied by a marked reduction in myocardial cardiolipin, particularly the tetralinoleoyl species 72:8 [6,7,8], which is essential for proper electron transport chain assembly and efficient mitochondrial respiration [7,9]. Cardiolipin deficiency destabilizes respiratory supercomplexes, impairs oxidative phosphorylation, and reduces energy availability, directly affecting diastolic function [10]. In a recent study, we demonstrated that 8 weeks of Empagliflozin treatment in an established experimental HFpEF model restored diastolic relaxation, accompanied by improved cardiac respiratory function and normalized expression levels of cardiolipin [11]. The mitochondria-targeted peptide Elamipretide (Ela) was originally developed as a mitochondria-targeted therapeutic for disorders characterized by mitochondrial dysfunction, including Barth syndrome, a rare genetic disorder of cardiolipin metabolism. Ela binds cardiolipin, thereby stabilizing the inner mitochondrial membrane and enhancing mitochondrial bioenergetics [12,13]. Preclinical studies have shown that Ela restores mitochondrial respiration, ATP production, and left ventricular function in experimental models of heart failure [12,14].

In human ex vivo studies, Ela improved mitochondrial oxygen flux and complex activities in the failing myocardium [6]. Clinical studies in heart failure with reduced ejection fraction (HFrEF) demonstrated safety and short-term improvements in mitochondrial function, although long-term cardiac outcomes remain inconclusive [15,16]. However, the results of a multicenter study evaluating Ela in HFpEF (Study ID: NCT 02814097) are still pending. Therefore, the present study aimed to assess whether Ela treatment of a preclinical model with established HFpEF leads to improved cardiac mitochondrial function and measurable cardiovascular benefits.

## 2. Results

### 2.1. Animal Characteristics

At 20 weeks of age and prior to intervention start, all ZSF1-obese rats (HFpEF and HFpEF/Ela) featured typical signs of HFpEF, including impaired diastolic function, preserved ejection fraction, and metabolic syndrome compared to age-matched lean control rats (Table A1). After 12 weeks of Ela treatment, neither body weight nor heart weight were different between HFpEF and HFpEF/Ela (Table 1).

### 2.2. Myocardial Performance

**Figure 1:**
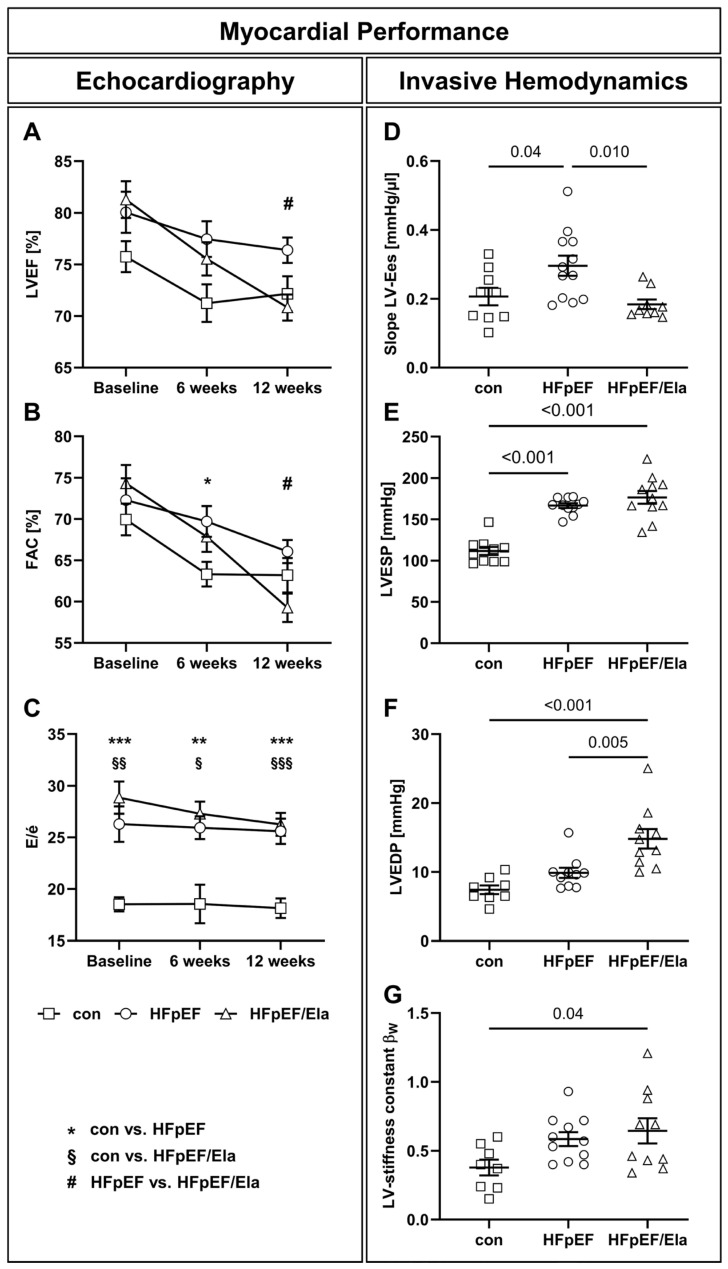
Myocardial performance displayed by echocardiographic and hemodynamic measurements. Left ventricular ejection fraction (LVEF, **A**) and fractional area change (FAC, **B**) were preserved in all groups at all times (charts show data after 12 weeks of Ela). Time course of diastolic function (**C**) showing that the ratios of early mitral inflow (E)/tissue Doppler mitral annulus early diastolic velocity (é) remained unchanged, with no improvement observed at any point during the entire experimental period. Invasive hemodynamic measurements revealed reduced myocardial contractility of HFpEF/Ela compared to untreated HFpEF (**D**). In contrast, left-ventricular end-systolic (LVESP, **E**) and end-diastolic pressures (LVEDP, **F**) were similarly high, or even further elevated in HFpEF/Ela relative to untreated HFpEF. LV stiffness was enhanced in heart failure with preserved ejection fraction (HFpEF) and further elevated in HFpEF/Ela (indicated by the stiffness constant β_W_, **G**); con denotes the lean control group; HFpEF denotes the obese control group; and HFpEF/Ela denotes the obese treatment group. LV-Ees, left ventricular end-systolic elastance. * *p* < 0.05, ** *p* < 0.01, *** *p* < 0.001: con vs. HFpEF; ^§^ *p* < 0.05, ^§§^ *p* < 0.01, ^§§§^ *p* < 0.001: con vs. HFpEF/Ela, ^#^ *p* < 0.05: HFpEF vs. HFpEF/Ela.

After 12 weeks of Ela treatment, left ventricular systolic function was slightly reduced in ZSF1-obese rats (Table 1, Figure 1). While left ventricular ejection fraction (LVEF) was preserved in all groups at all times, LVEF, fractional area change (FAC), and LV end-systolic elastance (Slope LV-Ees) declined modestly (Table 1, Figure 1A,B,D), accompanied by a small increase in end-systolic volume (LVESV, Table 1), indicating minor impairment of contractile performance. Diastolic function remained severely impaired, with persistently elevated E/é (Table 1, Figure 1C), left ventricular end-diastolic pressure (LVEDP, Figure 1E), and LV stiffness constant β_W_ (Figure 1F). Filling pressures showed a tendency to be higher under treatment conditions, with a significant increase in LVESP and numerically higher LVEDP and mean aortic pressure (Figure 1E, Table 1).

### 2.3. Cardiac Mitochondrial Function, Ultrastructure, and Lipid Remodeling

**Figure 2:**
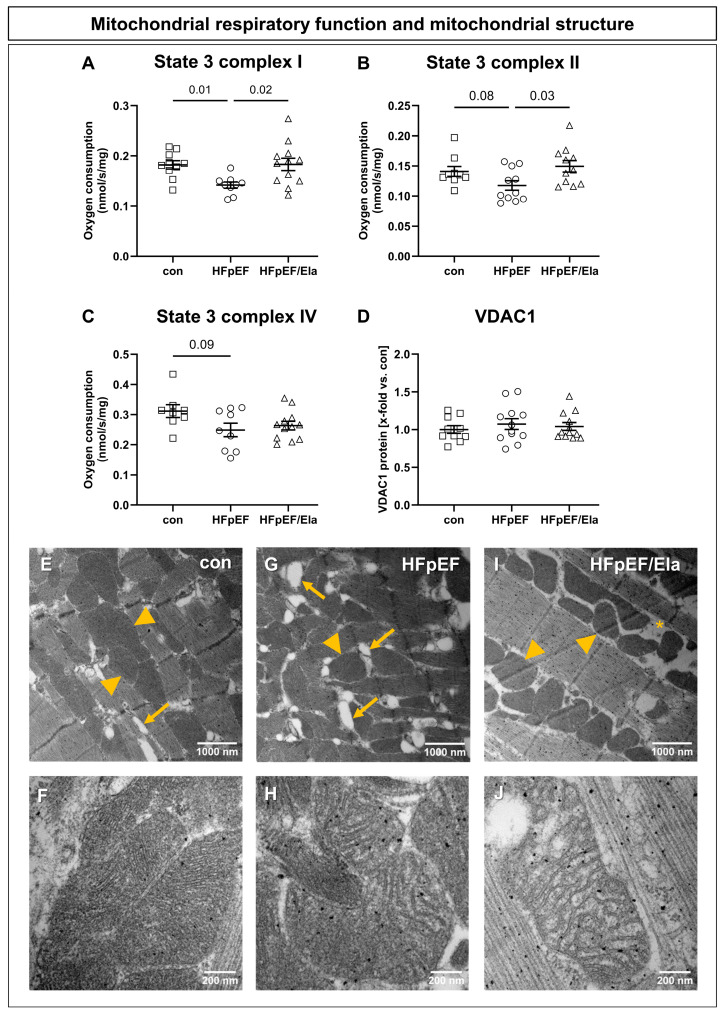
Impact of Ela treatment on mitochondrial respiratory function and structure. Stimulation of complex I with glutamate/malate (**A**) resulted in a reduced oxygen consumption in heart failure with preserved ejection fraction (HFpEF) compared with the control, whereas Ela (HFpEF/Ela) restored the oxygen consumption. Accordingly, stimulation of complex II with succinate (**B**) led to reduced oxygen consumption in HFpEF and significantly enhanced respiration rates in HFpEF/Ela. Complex IV-dependent oxygen consumption showed a tendency to reduce in HFpEF compared with con, whereas HFpEF/Ela remained comparable to HFpEF (**C**). Protein expression levels of VDAC1 (**D**), a marker for mitochondrial quantity, were similar between all groups. Electron micrographs depict intermyofibrillar mitochondria (yellow arrowheads) aligned with the contractile fibers in left-ventricular tissue of con (**E**,**F**), HFpEF (**G**,**H**), and HFpEF/Ela (**I**,**J**). In HFpEF, mitochondria displayed pronounced structural abnormalities, including disorganized cristae and increased lipid droplet content (yellow arrows, **G**,**H**). After Ela treatment, mitochondrial morphology appeared comparably impaired, with enlarged intermitochondrial spaces (asterisk) and cristae disruption (**I**,**J**); con denotes the lean control group; HFpEF denotes the obese control group; and HFpEF/Ela denotes the obese treatment group.

Ela treatment partially improved mitochondrial respiratory capacity in left ventricular muscle fibers of HFpEF/Ela (Figure 2). Complex I- and II-linked mitochondrial respiration was significantly increased compared to untreated HFpEF (Figure 2A,B), whereas Complex IV-dependent respiration remained unchanged (Figure 2C). VDAC1 abundance, serving as an indicator of mitochondrial content, was comparable among all three groups (Figure 2D), indicating that the observed functional differences were not attributable to altered mitochondrial density. Transmission electron microscopy revealed pronounced structural abnormalities of cardiac mitochondria in HFpEF, including disorganized and fragmented cristae and abundant lipid droplet accumulation between mitochondria (Figure 2G,H). Ela treatment did not improve the irregular mitochondrial morphology in HFpEF (Figure 2I,J).

**Figure 3:**
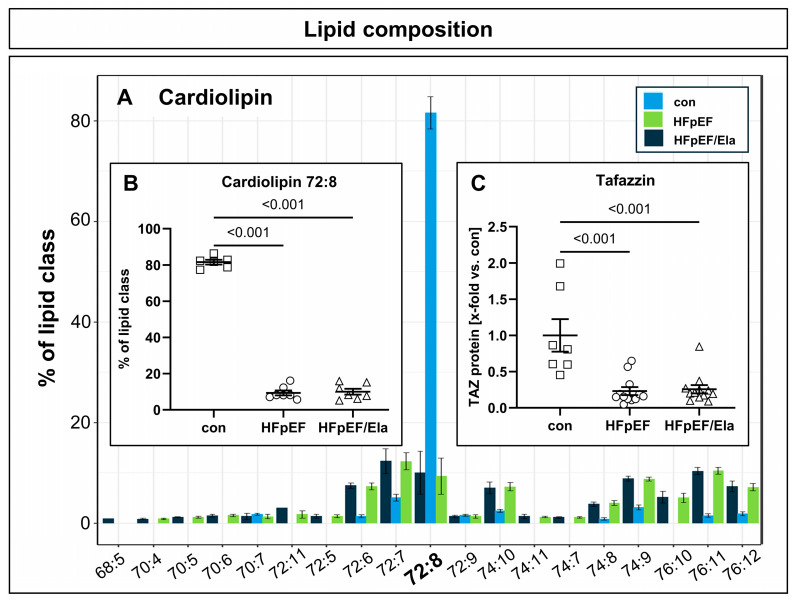
Impact of Ela treatment on left ventricular Cardiolipin concentration and maturation. Cardiolipin composition was profoundly altered in HFpEF, with tetralinoleoyl-cardiolipin (72:8) reduced from >80% in controls to <10% in both untreated and Ela-treated HFpEF hearts, indicating that Ela did not restore cardiolipin levels (**A**,**B**). Tafazzin (TAZ) protein expression was likewise markedly decreased in HFpEF and remained unchanged with Ela (**C**); con denotes the lean control group; HFpEF denotes the obese control group; and HFpEF/Ela denotes the obese treatment group.

Cardiolipin composition was markedly altered in HFpEF hearts (Figure 3A,B). In lean controls, tetralinoleoyl-cardiolipin (72:8) accounted for more than 80% of total cardiolipin species, whereas its relative abundance was reduced to below 10% in both untreated and treated HFpEF groups. Tafazzin protein expression was consistently strongly decreased in HFpEF hearts and remained unchanged after Ela treatment (Figure 3C).

### 2.4. Myocardial Remodeling and Inflammation

**Figure 4:**
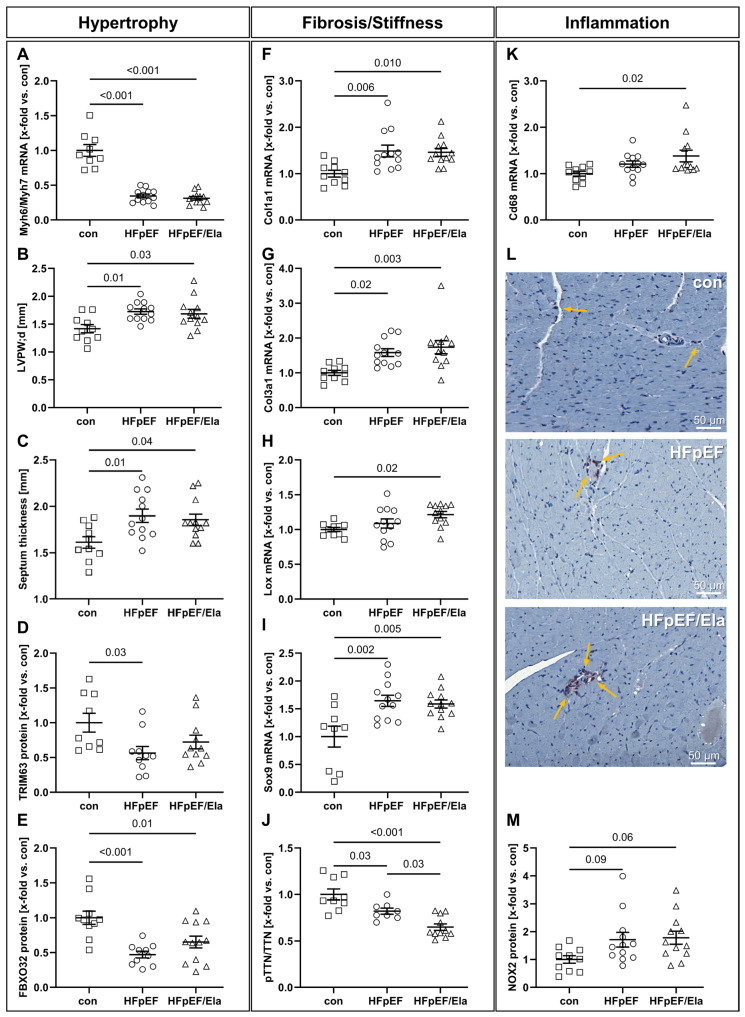
Impact of Ela treatment on left ventricular hypertrophy, fibrosis, and inflammation. Markers of cardiomyocyte hypertrophy were elevated in HFpEF and remained unchanged with Ela, including a reduced Myh6/Myh7 mRNA ratio (**A**), increased diastolic LV posterior wall (LVPW;d, **B**) and septum thickness (**C**). Protein levels of atrophy-related E3 ligases TRIM63 (**D**) and FBXO32 (**E**) were downregulated in HFpEF without normalization by Ela. Extracellular matrix remodeling was enhanced in HFpEF and not attenuated by treatment, as shown by increased mRNA expression levels of Col1a1 (**F**), Col3a1 (**G**), Lox (**H**), and Sox9 (**I**), along with reduced titin phosphorylation (**J**). Markers of inflammation were likewise elevated, with HFpEF/Ela showing further increases in Cd68 mRNA expression (**K**) and CD68^+^ macrophage clusters (**L**, yellow arrows), as well as upregulated Nox2 protein levels (**M**) in both HFpEF groups. TTN denotes titin and pTTN represents phosphorylated titin; con denotes the lean control group; HFpEF denotes the obese control group; and HFpEF/Ela denotes the obese treatment group.

Markers of cardiomyocyte hypertrophy were consistently elevated in HFpEF and remained unaffected by Ela (Figure 4A–E). The Myh6/Myh7 expression ratio, reflecting the balance between the fast α- and slow β-myosin heavy chain isoforms, was significantly reduced in both HFpEF groups compared with controls, a pattern commonly observed in hypertrophic remodeling (Figure 4A). Both left ventricular posterior wall thickness (LVPW;d) and interventricular septal thickness were significantly increased, suggesting structural hypertrophy of the myocardium (Figure 4B,C). In line with this, protein expression of the atrophy-related E3 ubiquitin ligases TRIM63 and FBXO32 was markedly reduced in HFpEF and showed no normalization with Ela. Markers of extracellular matrix remodeling and myocardial stiffness were upregulated in HFpEF and not attenuated by Ela (Figure 4F–J). Expression of Col1a1 and Col3a1 was significantly increased in both HFpEF groups compared with controls, indicating enhanced interstitial fibrosis (Figure 4F,G). The lysyl oxidase (Lox) transcript, mediating collagen cross-linking, was elevated in both HFpEF groups and reached statistical significance in HFpEF/Ela (Figure 4H). In addition, expression of the transcription factor Sox9, associated with profibrotic and chondrocytic reprogramming, was significantly upregulated in both HFpEF groups (Figure 4I). Consistent with increased myocardial stiffness, titin phosphorylation was reduced in HFpEF and further decreased in Ela-treated animals (Figure 4J). Markers of myocardial inflammation were elevated in HFpEF and remained increased under Ela treatment conditions (Figure 4K–M). Expression of the macrophage marker Cd68 was significantly higher in Ela-treated hearts compared to the lean control (Figure 4K) and was accompanied by the appearance of clustered CD68-positive cell nests in immunohistochemical sections, indicating enhanced macrophage accumulation in both obese groups (Figure 4L). In addition, protein expression of NADPH oxidase 2 (NOX2), a key source of reactive oxygen species, was significantly increased in both HFpEF groups compared with controls (Figure 4M).

### 2.5. Carotid Vascular Function, Stiffness, and Molecular Profile

**Figure 5:**
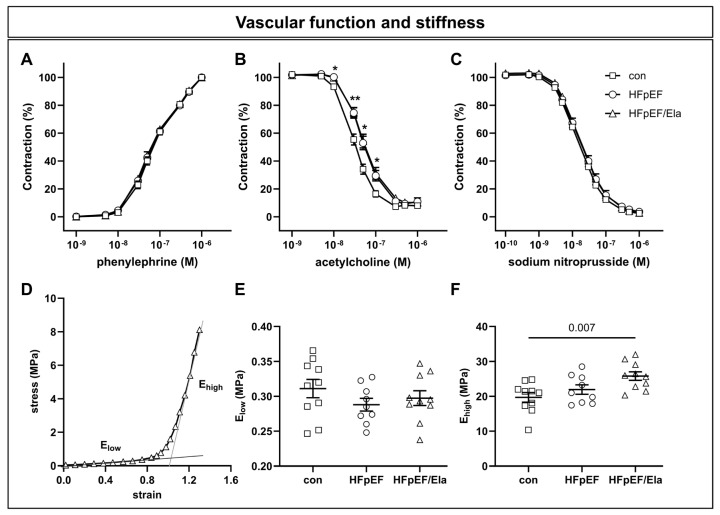
Impact of Ela treatment on carotid vascular function and stiffness. Endothelium-dependent relaxation mediated by acetylcholine was impaired in HFpEF and not improved by Ela (**A**), while phenylephrine-induced contraction (**B**) and sodium nitroprusside-mediated relaxation (**C**) were comparable across groups. Vascular stiffness assessment (exemplary stress–strain curve, **D**) revealed unchanged elastin-dependent elasticity (E_low_, **E**), whereas collagen-dependent stiffness (E_high_, **F**) was increased in HFpEF/Ela compared with controls but not relative to HFpEF; con denotes the lean control group; HFpEF denotes the obese control group; and HFpEF/Ela denotes the obese treatment group. * *p* < 0.05 and ** *p* < 0.01 HFpEF vs. con.

Vascular function in carotid arteries was assessed to evaluate endothelial and smooth muscle performance (Figure 5). Evaluation of endothelium-dependent relaxation using acetylcholine confirmed the previously reported impaired relaxation of HFpEF compared to con animals (Figure 5A) [17]. Treatment with Ela did not result in any improvement. Neither the phenylephrine-induced contraction (Figure 5B) nor the sodium nitroprusside-induced, endothel-independent relaxation (Figure 5C) differed among the experimental groups.

Vascular stiffness was measured ex vivo in carotid segments. Two distinct elastic moduli (E_low_ and E_high_) were calculated from the stress–strain curves (Figure 5D). E_low_ corresponds to the elastic modulus of the low-strain region and reflects elastin-mediated elasticity. E_high_ is derived from the high-strain region and indicates collagen-dependent arterial stiffness. E_low_ was similar across all groups (Figure 5E), whereas E_high_ was elevated in HFpEF/Ela animals compared to controls but not versus HFpEF animals (Figure 5F).

To investigate the structural basis of this increase in stiffness, carotid morphology was examined using Elastica van Gieson staining and morphometric analysis (Table 2). No differences were observed in media thickness, internal circumference, number of elastic laminae, or thickness of smooth muscle layers between laminae across groups. Consistent with these findings, mRNA expression of genes associated with extracellular matrix composition was largely unchanged (Table 2). The only exception was Lox, which was modestly upregulated in the HFpEF/Ela versus the HFpEF animals.

## 3. Discussion

In our study, treatment with Ela in established preclinical HFpEF partially improved complex I- and II-linked mitochondrial respiration but failed to produce functional, structural, or vascular benefits. The major findings are as follows: (1) enhanced mitochondrial respiration without improvement in diastolic function; (2) persistent mitochondrial ultrastructural defects and unchanged cardiolipin content; (3) unaltered cardiac remodeling and slightly increased inflammation; and (4) unaffected endothelial dysfunction with no improvement in vascular mechanical properties.

### 3.1. Cardiac Performance and Hemodynamics

Despite the observed improvement in mitochondrial respiration, Ela treatment did not translate into measurable benefits in cardiac performance. Diastolic dysfunction remained severe, as indicated by persistently elevated E/é, LVEDP, and LV stiffness constant β_W_ while systolic function modestly declined. Hemodynamic parameters further reflected this lack of improvement: LVESP was significantly increased under treatment conditions, and MAP tended to be higher, while LVEDP remained numerically elevated. These findings suggest that mitochondrial bioenergetic enhancement alone is insufficient to improve diastolic relaxation, or cardiac hemodynamics in established HFpEF.

Previous studies in metabolic HFpEF models suggest that mitochondrial-targeted therapies are capable of ameliorating diastolic dysfunction when administered before the onset of extensive structural remodeling [18]. In contrast, our data demonstrate that once HFpEF is fully established, diastolic relaxation as well as hemodynamic parameters are largely unresponsive to Ela, highlighting the importance of timing in therapeutic interventions. Moreover, the observed increase in blood pressure and modest decline in systolic function may even indicate potential adverse effects of Ela in this setting.

### 3.2. Mitochondrial Ultrastructure and Cardiolipin Profile

Although Ela improved complex I- and II-linked respiration in cardiac fibers, mitochondrial ultrastructure showed further disruption, including fragmented cristae and increased inter-mitochondrial spacing. Neither cardiolipin composition nor quantity were altered by Ela, which is in line with results of Ela treatment of nonfailing and failing human myocardial samples [6]. Direct evidence on mitochondrial ultrastructure and cardiolipin remodeling in human HFpEF is limited. Available studies using human cardiac samples predominantly focus on HFrEF and report a marked reduction in total cardiolipin levels, particularly tetralinoleoyl-cardiolipin (CL 72:8), which is associated with impaired mitochondrial function [6,7,8]. In response to Ela treatment, mitochondrial oxygen flux and complex activity were significantly improved, likely through improved coupling of the mitochondrial supercomplex [6]. Notably, Ela improved respiration in intact mitochondria isolated from failing human hearts, suggesting that its effect may be restricted to relatively preserved mitochondria and limited by the severity of mitochondrial damage. In our study, particularly CL 72:8, remained depleted (<10% of total cardiolipin), and expression of tafazzin, a crucial enzyme involved in cardiolipin maturation, was unchanged. These findings suggest that functional effects of Ela are insufficient to reverse structural and compositional mitochondrial defects once HFpEF is established.

Cardiolipin is essential for inner mitochondrial membrane integrity, supercomplex assembly, and efficient oxidative phosphorylation [19]. Studies in tafazzin-deficient models demonstrate that severe cardiolipin depletion results in disorganized cristae, impaired electron transport, and cardiac dysfunction [20]. Allen et al. demonstrated improved mitochondrial respiration and mitigated cristae fragmentation by Ela in acute ischemia–reperfusion, even though cardiolipin content remained reduced in the treatment group [21]. In addition, the duration of Ela exposure may be a critical factor. Three months of elamipretide treatment reversed abnormalities in mitochondrial dynamics in left ventricular tissue of dogs and humans with HFrEF, including restoration of cardiolipin content [22]. On the other hand, four weeks of Ela treatment in patients with stable HFrEF were well tolerated but did not improve LVESV [16]. However, this study did not report cardiolipin levels. In studies investigating Ela treatment in Barth syndrome, a genetic disorder of mitochondrial cardiolipin metabolism, associated with an HFpEF-like cardiomyopathy (reduced left ventricular volumes, preserved ejection fraction, and impaired active and passive relaxation and filling), functional improvements were only observed after long-term treatment (168 weeks) [23]. These improvements were correlated with an increased ratio of mature to immature cardiolipin species. In contrast, a study with shorter treatment durations (12 weeks) reported that this ratio remained at baseline levels and failed to meet primary and secondary endpoints, including myocardial functional parameters [24]. These findings suggest that short-term improvements in mitochondrial function may precede, but are not sufficient to drive, structural remodeling or physiological recovery, particularly in advanced disease states such as HFpEF. In our chronic HFpEF model, cardiolipin content remained downregulated, but in contrast to the HFrEF data, partial enhancement of mitochondrial respiration did not prevent further mitochondrial ultrastructural deterioration, indicating that protective effects might not translate into effective rescue in established HFpEF [21].

### 3.3. Cardiac Remodeling and Inflammation

Ela did not ameliorate hypertrophy, fibrosis, or inflammation. The Myh6/Myh7 ratio remained reduced, posterior wall and septal thicknesses were increased, titin phosphorylation was further decreased, and E3 ubiquitin ligases TRIM63 and FBXO32 remained suppressed. Fibrotic and inflammatory markers were unaltered or even slightly increased.

Persistent structural and inflammatory remodeling likely limits the translation of mitochondrial improvement into functional benefit. Fibrosis and titin hypophosphorylation are established contributors to diastolic stiffness in HFpEF [17,25,26]. Similarly, macrophage infiltration and ROS overproduction exacerbate mitochondrial dysfunction and ECM remodeling, forming a reinforcing pathogenic loop [11,27,28].

Previous studies suggest that early targeting of mitochondrial dysfunction can attenuate hypertrophy and fibrosis [29]. Our data extend these findings, demonstrating that once maladaptive remodeling is established, Ela alone cannot reverse hypertrophy, fibrosis, or inflammation. This supports the notion that HFpEF is a multi-factorial syndrome where mitochondrial dysfunction is only one contributor among systemic metabolic, inflammatory, and fibrotic pathways.

### 3.4. Vascular Function and Stiffness

Vascular function remained largely unaltered under Ela treatment conditions. Endothelium-dependent relaxation was not improved by Ela, and ex vivo measurements of elastin-mediated elasticity (E_low_) were unaffected. Minor increases in collagen-dependent stiffness (E_high_) were observed compared with control rats but did not reach significance relative to untreated HFpEF.

Vascular remodeling, endothelial dysfunction, and stiffening are recognized hallmarks of HFpEF and closely linked to systemic inflammation and ECM remodeling [17,30]. Our findings suggest that mitochondrial-targeted therapy alone does not sufficiently address these vascular abnormalities, further limiting potential functional benefits.

### 3.5. Clinical Implications

In our study, the dissociation between improved mitochondrial respiration and the absence of functional or structural benefit suggests that targeting mitochondrial bioenergetics alone may not be sufficient in established HFpEF. Our findings align with clinical studies of Ela in HFrEF and Barth syndrome, where treatment durations of only 1–3 months did not result in measurable improvements in cardiac performance [16,24]. Overall, Ela was generally well tolerated. Reported adverse events were predominantly mild to moderate in severity and were not considered treatment-related [16,23,24]. No clinically relevant changes were observed in key cardiovascular parameters, including left ventricular ejection fraction and blood pressure. Considering these clinical observations [15], our results suggest the need for combinatorial approaches in HFpEF treatment. Strategies that concurrently target inflammation, fibrosis, and metabolic dysregulation (e.g., SGLT2 inhibitors or GLP-1 RA) may offer greater potential than mitochondrial therapy alone. Moreover, timing appears critical: early intervention before extensive remodeling may be necessary to achieve effective functional outcomes.

### 3.6. Limitations

While the ZSF1-obese rat model reliably recapitulates metabolic HFpEF features [31], species differences and the timing of intervention limit direct translation to humans. Furthermore, only female animals were used in this study. The Ela dose and duration were chosen based on prior studies but may not fully reflect clinical pharmacokinetics. Functional endpoints in rodents may not capture subtle hemodynamic or exercise capacity improvements relevant to patients. The present study did not include direct measurements of mitochondrial ATP production, coupling efficiency (P/O ratio), or mitochondrial ROS emission. While mitochondrial respiration was comprehensively assessed, inclusion of these additional parameters will be important in future studies to further refine the interpretation of mitochondrial efficiency and redox balance in HFpEF.

## 4. Materials and Methods

### 4.1. Animals

Female ZSF1-lean and ZSF1-obese rats were used as an established preclinical model of heart failure with preserved ejection fraction (HFpEF). All animals were obtained from Charles River Laboratories (Sulzfeld, Germany). Upon arrival, rats were acclimatized for at least one week before any experimental procedures were performed.

At study start, animals were 20 weeks of age. A total of 10 ZSF1-lean rats and 24 ZSF1-obese rats were included in the study. Baseline transthoracic echocardiography was performed in all animals at 20 weeks of age to confirm the presence of HFpEF-related cardiac alterations in obese rats and to exclude animals with unrelated cardiac abnormalities. No animals were excluded after baseline assessment.

Following baseline echocardiography, ZSF1-obese rats were randomly allocated to one of two experimental groups using a computer-generated randomization scheme: a vehicle-treated HFpEF group (HFpEF, *n* = 12) or an Ela-treated group (HFpEF/Ela, *n* = 12). Animals were assigned continuous identification numbers independent of group allocation, and experimental procedures and measurements were performed according to these identifiers to minimize potential confounders related to measurement order. Investigators performing echocardiographic and invasive hemodynamic assessments were blinded to group allocation, with animals identified solely by continuous identification numbers. ZSF1-lean rats served as age-matched healthy controls (con, *n* = 10) and did not receive any pharmacological treatment.

Ela was administered at a dose of 3 mg/kg/day via subcutaneously implanted osmotic minipumps (Alzet^®^, DURECT Corporation, Cupertino, CA, USA), while vehicle-treated animals received pumps filled with the corresponding vehicle solution. Minipump implantation was performed under general anesthesia (isoflurane), and animals received appropriate perioperative care according to institutional guidelines.

Echocardiographic assessments were repeated after 6 and 12 weeks of treatment to monitor cardiac structure and function longitudinally. At the end of the 12-week treatment period, invasive hemodynamic measurements of left ventricular and aortic function were performed under anesthesia, followed by tissue collection for further analyses.

All experimental procedures were conducted in accordance with national and institutional regulations for animal welfare and were approved by the responsible local animal ethics committee.

### 4.2. Echocardiography

Rats were anesthetized with isoflurane (1.5–2%) and placed on a temperature-controlled warming pad to maintain physiological body temperature. Transthoracic echocardiography was performed using a Vevo 3100 system equipped with a 21 MHz transducer (FUJIFILM VisualSonics Inc., Amsterdam, The Netherlands) to assess cardiac structure and function. Systolic function was evaluated from parasternal long- and short-axis views at the level of the papillary muscles using B-mode and M-mode imaging. Diastolic function was assessed in the apical four-chamber view using pulsed-wave Doppler to measure early (E) and atrial (A) transmitral inflow velocities, and tissue Doppler imaging was employed to quantify early myocardial relaxation velocity (é) at the basal septal segment of the left ventricle. Functional parameters, including LVEF, stroke volume, and E/é, were derived using Vevo LAB 3.2.6 software.

### 4.3. Invasive Hemodynamics

Prior to organ harvest, invasive left ventricular hemodynamics were assessed as previously described [17] in anesthetized, spontaneously breathing rats (ketamine 105 mg/kg and xylazine 7 mg/kg, i.p.). For pressure–volume (PV) analysis, the right carotid artery was cannulated with a rat PV catheter (SPR-838, ADInstruments Ltd., Oxford, UK), which was advanced into the mid–left ventricular cavity. PV loops were recorded under baseline conditions and during transient occlusion of the inferior vena cava by gentle external compression to obtain load-independent indices of contractility and chamber stiffness. End-systolic and end-diastolic pressure–volume relationships (ESPVRs and EDPVRs) were fitted to linear and exponential functions, respectively, with the slope of the ESPVR (Ees) indicating contractility and the chamber stiffness constant (β) reflecting diastolic compliance. To account for differences in cardiac size between groups, left ventricular wall volume (V_w_) was used as a normalization factor (β × V_w_ = β_w_), as previously reported [17,32,33]. Data acquisition and analysis were performed using LabChart 8 software (ADInstruments Ltd., Oxford, UK).

### 4.4. Carotid Artery Function

Measurement of vascular function was performed on carotid artery rings, which were cut into approximately 3 mm segments and mounted in duplicate on 200 µm pins of the multi-channel wire myograph (630MA, Danish Myo Technology, Aarhus, Denmark). An organ bath was filled with Krebs–Henseleit buffer (118 mmol/L NaCl, 25 mmol/L NaHCO_3_, 4.7 mmol/L KCl, 1.2 mmol/L KH_2_PO_4_, 1.2 mmol/L MgSO_4_, 2.5 mmol/L CaCl_2_, 5.5 mmol/L Glucose, pH 7.4) and gassed with carbogen, and the temperature was kept at 37 °C. Segments were equilibrated without any tension for 15 min. The optimal preconstriction was determined by measuring force responses after stimulation with KCl and phenylephrine in preliminary experiments. Each vessel ring was set up to the same initial conditions by stepwise stretching to an effective pressure of 10 kPa (75 mmHg) as calculated using the Laplace equation: Effective Pressure = Wall Tension/(Internal Circumference/2π). Maximal contraction was assessed by adding KCl (final concentration 0.1 mol/L). Phenylephrine (PE)-dependent contraction was determined by adding PE up to a concentration of 1 × 10^−6^ mol/L. Relaxation measurements were conducted after PE contraction (3 × 10^−7^ mol/L) with increasing concentrations of acetylcholine (ACh Sigma, Rödermark, Germany; 1 × 10^−9^ to 1 × 10^−6^ mol/L) and sodium nitroprusside (SNP; Sigma, Rödermark, Germany; 1 × 10^−10^ to 1 × 10^−6^ mol/L). Vessel tension was normalized to vessel length, and contractions were calculated as percentages.

Ex vivo assessment of arterial mechanics was conducted as previously described [17,34,35,36]. Briefly, the left carotid artery was isolated, cleared of surrounding connective tissue, and prepared for analysis of intrinsic wall stiffness. Two segments of approximately 0.5 mm were obtained, and unloaded geometric parameters (internal circumference, wall thickness, and segment length) were determined from microscopic images. The rings were mounted on 200 µm wires in temperature-controlled myograph chambers (Danish Myo Technology, Aarhus, Denmark) filled with calcium- and magnesium-free PBS. After an equilibration period of at least 30 min, vessels underwent two standardized prestretching cycles to ~5 mN, each followed by a short resting phase. Subsequently, the system was zeroed, and rings were gently stretched until a minimal force of ~0.5 mN was reached. Incremental stretching steps were then applied every 3 min (5–10% of the unloaded internal circumference). Force and displacement were continuously recorded until 400 mN was exceeded or structural failure occurred. Strain (ε = (r − r_0_)/r_0_) and circumferential stress (σ_c_ = (F·r)/(2·L·h·r_0_)) were calculated using unloaded radius (r_0_), current radius (r), wall thickness (h), and segment length (l). The elastic moduli (E_low_ and E_high_) were derived from the slopes of the stress–strain curve.

### 4.5. Left Ventricular Mitochondrial Respiration

Mitochondrial respiratory capacity was quantified in saponin-permeabilized fibers obtained from the left ventricle, following established procedures [11]. Oxygen consumption was recorded at 25 °C using a Clark-type electrode (Strathkelvin Instruments, Motherwell, UK) in a stirred oxygraphic chamber. To exclude diffusion limitations, the oxygen concentration in the chamber was initially raised to ~400 µmol/L and maintained above 270 µmol/L during all measurements. Left ventricular fibers were prepared in a permeabilization buffer (SolP) containing (mmol/L): 2.77 CaK_2_EGTA, 7.23 K_2_EGTA, 6.56 MgCl_2_, 5.7 Na_2_ATP, 15 phosphocreatine, 20 taurine, 0.5 DTT, 50 K-methanesulfonate, and imidazole (pH 7.1). Samples were incubated for 30 min with saponin (50 µg/mL) and subsequently transferred for 10 min into a respiration buffer (SolR; mmol/L: 20 taurine, 20 HEPES, 10 KH_2_PO_4_, 0.5 EGTA, 3 MgCl_2_, 0.11 sucrose, 60 K-lactobionate; pH 7.4) at 4 °C to remove endogenous nucleotides and phosphocreatine. For measurements, 1–5 mg of permeabilized fibers were placed in 1 mL SolR supplemented with 1 mg/mL BSA. A sequential substrate–inhibitor protocol was used to characterize distinct respiratory states: (I) glutamate (10 mmol/L) + malate (2 mmol/L) for basal complex I activity; (II) ADP (5 mmol/L) for complex I-supported oxidative phosphorylation; (III) octanoylcarnitine (0.2 mmol/L) to assess FAO-driven respiration; (IV) cytochrome c (10 µmol/L) to evaluate mitochondrial outer membrane integrity; (V) succinate (10 mmol/L) for combined complex I+II respiration; (VI) rotenone (0.5 µmol/L) for complex II-specific flux; (VII) FCCP (0.5 µmol/L) for maximal uncoupled respiration; (VIII) antimycin A (2.5 µmol/L) to inhibit complex III; and (IX) ascorbate (2 mmol/L) + TMPD (0.5 mmol/L) for complex IV capacity. After completion of the protocol, fiber bundles were briefly blotted and weighed. Respiration is expressed as nmol O_2_·s^−1^·mg^−1^ wet tissue.

### 4.6. Lipid Extraction for Mass Spectrometry Lipidomics

Mass spectrometry-based lipid analysis was performed by Lipotype GmbH (Dresden, Germany) as described [37]. Lipids were extracted using a chloroform/methanol procedure [38]. Samples were spiked with internal lipid standard mixture containing cardiolipin 14:0/14:0/14:0/14:0 (CL), ceramide 18:1;2/17:0 (Cer), diacylglycerol 17:0/17:0 (DAG), hexosylceramide 18:1;2/12:0 (HexCer), lyso-phosphatidate 17:0 (LPA), lyso-phosphatidylcholine 12:0 (LPC), lyso-phosphatidylethanolamine 17:1 (LPE), lyso-phosphatidylglycerol 17:1 (LPG), lyso-phosphatidylinositol 17:1 (LPI), lyso-phosphatidylserine 17:1 (LPS), phosphatidate 17:0/17:0 (PA), phosphatidylcholine 15:0/18:1 D7 (PC), phosphatidylethanolamine 17:0/17:0 (PE), phosphatidylglycerol 17:0/17:0 (PG), phosphatidylinositol 16:0/16:0 (PI), phosphatidylserine 17:0/17:0 (PS), cholesterol ester 16:0 D7 (CE), sphingomyelin 18:1;2/12:0;0 (SM), and triacylglycerol 17:0/17:0/17:0 (TAG). After extraction, the organic phase was transferred to an infusion plate and dried in a speed vacuum concentrator. The dry extract was re-suspended in 7.5 mM ammonium formiate in chloroform/methanol/propanol (1:2:4; V/V/V). All liquid handling steps were performed using the Hamilton Robotics STARlet robotic platform with the Anti Droplet Control feature for pipetting organic solvents.

### 4.7. MS Data Acquisition

Samples were analyzed by direct infusion on a QExactive mass spectrometer (Thermo Scientific, Waltham, MA, USA) equipped with a TriVersa NanoMate ion source (Advion Biosciences, Ithaca, NY, USA). Samples were analyzed in both positive and negative ion modes with a resolution of R_m/z=200_ = 280,000 for MS and R_m/z=200_ = 17,500 for MSMS experiments, in a single acquisition. MSMS was triggered by an inclusion list encompassing corresponding MS mass ranges scanned in 1 Da increments [39]. Both MS and MSMS data were combined to monitor CE, DAG, and TAG ions as ammonium adducts; LPC, LPC O-, PC, and PC O- as formiate adducts; and CL, LPS, PA, PE, PE O-, PG, PI, and PS as deprotonated anions. MS was only used to monitor LPA, LPE, LPE O-, LPG, and LPI as deprotonated anions and Cer, HexCer, and SM as formiate adducts.

### 4.8. Data Analysis and Post-Processing

Data were analyzed with in-house-developed lipid identification software based on the LipidXplorer (https://lifs-tools.org/tools/lipidxplorer, accessed on 16 December 2025) [40,41]. Data post-processing and normalization were performed using an in-house-developed data management system. Only lipid identifications with a signal-to-noise ratio > 5 and a signal intensity 5-fold higher than in corresponding blank samples were considered for further data analysis.

### 4.9. Titin Analysis

Assessment of titin expression and phosphorylation was performed using vertical agarose gel electrophoresis (VAGE) as recently described [42]. Snap-frozen soleus muscle was pulverized and extracted in urea buffer (8 mol/L urea, 2 mol/L thiourea, 0.05 mol/L Tris pH 6.8, 0.075 mol/L DTT, 3% SDS) supplemented with phosphatase and protease inhibitors (Serva, Heidelberg, Germany) at a 1:50 (*w*/*v*) ratio. Samples were gently mixed by inversion and incubated at 60 °C for 10 min to ensure complete solubilization, followed by brief centrifugation (30 s, 4000× *g*). Protein concentration of the supernatant was determined using the 660 nm assay (Thermo Fisher Scientific, Waltham, MA, USA). Glycerol containing traces of bromophenol blue was added to a final concentration of 25%, and samples were centrifuged again (10 min, 13,200× *g*). Supernatants were aliquoted and stored at −80 °C.

For VAGE, 4–6 µg of protein was loaded onto 1% agarose gels and separated at 15 mA for 5 h as described by Zhu and Guo [43]. Phosphorylated titin was visualized using Pro-Q™ Diamond Phosphoprotein Gel Stain (Thermo Fisher Scientific, Waltham, MA, USA), followed by Sypro Ruby staining to assess total titin expression (Thermo Fisher Scientific). Titin phosphorylation was normalized to the corresponding non-phosphorylated titin expression.

### 4.10. Western Blot Analysis

For Western blot analyses, snap-frozen left ventricular tissue was homogenized in Relax buffer (90 mmol/L HEPES, 126 mmol/L potassium chloride, 36 mmol/L sodium chloride, 1 mmol/L magnesium chloride, 50 mmol/L EGTA, 8 mmol/L ATP, 10 mmol/L creatine phosphate; pH 7.4) supplemented with a protease inhibitor mix (Inhibitor mix M, Serva, Heidelberg, Germany) and sonicated. Protein concentration was determined using a BCA assay (Pierce, Bonn, Germany), and aliquots of 5–20 µg were separated by SDS-PAGE. Proteins were transferred to PVDF membranes and incubated overnight at 4 °C with the following primary antibodies: VDAC1 (1:1000, Proteintech, Planegg-Martinsried, Germany), tafazzin (1:500, Santa Cruz, Dallas, TX, USA), TRIM63 (1:200, Santa Cruz, Dallas, TX, USA), FBXO32 (1:1000, Abcam, Cambridge, UK), and Nox2 (1:1000, Abcam, Cambridge, UK). Membranes were then incubated with horseradish peroxidase-conjugated secondary antibodies, and specific bands were visualized by enhanced chemiluminescence (SuperSignal West Pico, Thermo Fisher Scientific Inc., Bonn, Germany). Densitometric quantification was performed using 1D Scan software (version: v15.08b, Scanalytics Inc., Rockville, MD, USA). Signals were normalized to GAPDH (1:30,000; HyTest Ltd., Turku, Finland). All data are presented as x-fold vs. control.

### 4.11. RNA Isolation and Real-Time RT-PCR

Total RNA was extracted from left ventricular tissue using Qiazol reagent and the miRNeasy Mini Kit (Qiagen, Hilden, Germany) according to the manufacturer’s instructions. cDNA synthesis was performed with the RevertAID™ H Minus First Strand Synthesis Kit (Thermo Scientific, Braunschweig, Germany) using oligo-dT primers. Quantitative real-time PCR was carried out on a CFX384™ Real-Time PCR System (Bio-Rad Laboratories GmbH, Feldkirchen, Germany) using the Maxima SYBR Green qPCR Kit (Thermo Scientific). The cycling protocol for all primer sets consisted of an initial denaturation at 95 °C for 8 min, followed by 40 cycles of 95 °C for 10 s, 58 °C for 15 s, and 72 °C for 30 s, with a final extension at 72 °C for 2 min. Melt curve analysis confirmed specificity of the amplified products. Relative mRNA expression levels were calculated using the ΔΔCT method with Polr2a and Tbp as reference genes, employing Bio-Rad CFX Manager Software, version 5.3.22.1030. All data are expressed in arbitrary units (AU), with values for the con group normalized to 1. Primer sequences are provided in Table A2.

### 4.12. Immunohistochemistry

Paraffin-embedded heart sections (3 μm) were stained with rabbit-polyclonal anti-CD68 (ab125212, Abcam, Berlin, Germany) using standard protocols as recently described [17,44].

Immunohistochemical staining was carried out using the Dako EnVision^®^+ System–HRP (AEC) (Dako, Jena, Germany). Target retrieval was performed by boiling with appropriate buffer and subsequent slow cooling for 30 min. Endogenous peroxidase activity was quenched with peroxidase block (Dako, Germany) for 10 min. Incubation with protein blocking solution (Dako, Jena, Germany) was performed for 20 min, and sections were subsequently incubated with rabbit-polyclonal anti-CD68 (ab125212, Abcam, Berlin, Germany) diluted in antibody diluent solution (Dako, Germany) for 1 h. Thereafter an anti-rabbit antibody conjugated to HRP-labeled polymer was applicated for 45 min. Color development was carried out with AEC+ substrate (Dako, Germany). Subsequently all slides were counterstained with Mayer’s hemalum and mounted with Protaqs Mount Flour (Quartett, Berlin, Germany). Negative controls were performed by omitting the primary antibodies. Slides were scanned using an Axio Scan.Z1 slide scanner (Carl Zeiss AG, Oberkochen, Germany) at the Light Microscopy Facility, CMCB Dresden.

### 4.13. Transmission Electron Microscopy

For transmission electron microscopy, samples from the three experimental conditions were chemically fixed in 2% (*v*/*v*) glutaraldehyde (EMS, Hatfield, PA, USA) in 0.1% phosphate buffer (pH 7.4) immediately after dissection. Post-fixation was performed with 1% (*w*/*v*) osmium tetroxide (EMS, USA) in double-distilled water. After three washing steps, tissues were dehydrated through a graded ethanol series (70, 80, 90, 96, and twice 100%; 30 min each) and infiltrated with Epon/Araldite (EMS, USA) resin (resin/ethanol ratios of 1:3 for 3.5 h, 1:1 overnight, and 3:1 for 3.5 h), followed by two steps in 100% resin (1 h and overnight). Samples were then embedded in block molds and polymerized at 60 °C for 48 h. Ultrathin sections (70 nm) were cut on an ultramicrotome (EM UC6, Leica Microsystems, Vienna, Austria) equipped with a diamond knife (Diatome, Biel/Bienne, Switzerland). Sections were mounted on Formvar-coated copper slot grids and post-stained with 2% (*w*/*v*) uranyl acetate in 70% methanol for 10 min, followed by 0.4% (*w*/*v*) lead citrate (EMS, Hatfield, PA, USA) for 5 min. Imaging was performed using a Morgagni transmission electron microscope (Thermo Fisher Scientific, Waltham, MA, USA) operated at 80 kV and equipped with a 2k × 2k CCD camera (Veleta, EMSIS, Münster, Germany).

### 4.14. Statistical Analyses

The sample size was determined a priori based on a statistical power calculation. The calculation was performed assuming a two-sided significance level of 0.05 and a statistical power of 80%, based on expected treatment effects derived from previous studies in the ZSF1 HFpEF model.

All data are expressed as mean ± SEM (standard error of the mean). One-way analysis of variance (ANOVA) followed by Bonferroni post hoc was used to compare all three groups of the treatment study. Two-way repeated measures ANOVA followed by Bonferroni post hoc was used to compare data from echocardiography and vascular function measurements between the groups over time and to assess contractile function. *p*-values below 0.05 were considered to be statistically significant.

## 5. Conclusions

In established preclinical HFpEF, Ela enhanced mitochondrial respiration but did not improve cardiac function, reverse maladaptive remodeling, normalize cardiolipin content, or confer vascular benefits. These findings suggest that mitochondrial-targeted therapy alone is insufficient once HFpEF is fully established, emphasizing the need for multi-targeted approaches and early intervention strategies.

## Figures and Tables

**Table 1:** Animal characteristics after 12 weeks of treatment retrieved from physiological and morphometric measurements, echocardiography, and invasive hemodynamic measurements.

***Physiology***			
**Parameter**	**con (*n* = 10)**	**HFpEF (*n* = 12)**	**HFpEF/Ela (*n* = 12)**
Organs			
Body weight [g]	270 ± 3	525 ± 8 ***	534 ± 8 ***
Tibia length [TL, mm]	35.4 ± 0.1	35 ± 0.1 **	35.4 ± 0.1 ^#^
LV weight/TL [mg/mm]	19.2 ± 1.1	27.5 ± 1.1 ***	26.2 ± 1.1 ***
Lung wet weight/TL [mg/mm]	10.9 ± 0.2	12.4 ± 0.3 **	12.4 ± 0.6 *
Kidney weight/TL [mg/mm]	28.3 ± 0.5	46.6 ± 1.2 ***	46.3 ± 1.9 ***
***Echocardiography***			
**Parameter**	**con (*n* = 10)**	**HFpEF (*n* = 12)**	**HFpEF/Ela (*n* = 12)**
LV mass [mg]	613 ± 28	931 ± 23 ***	925 ± 46 ***
LVEF [%]	72.2 ± 2	76.4 ± 1	70.8 ± 1 ^#^
FAC [%]	63.2 ± 2	66.1 ± 1 *	59.3 ± 2 ^#^
LVFS [%]	24 ± 2	31 ± 2 *	29 ± 1
LVSV [µL]	272 ± 16	380 ± 22 **	358 ± 26 *
LVEDV [µL]	376 ± 18	496 ± 26 *	500 ± 35 *
E/é	18.1 ± 0.9	25.6 ± 1.2 ***	26.3 ± 1.1 ***
E/A	1.8 ± 0.2	1.6 ± 0.1	1.5 ± 0.1
LVPW;d [mm]	1.4 ± 0.1	1.7 ± 0.1 *	1.7 ± 0.1 *
Septum;d [mm]	1.6 ± 0.1	1.9 ± 0.1 *	1.9 ± 0.1 *
LVID;d [mm]	7.8 ± 0.2	9.0 ± 0.2 ***	9.1 ± 0.2 ***
***Invasive Hemodynamics***			
**Parameter**	**con (*n* = 8**–**10)**	**HFpEF (*n* = 10**–**12)**	**HFpEF/Ela (*n* = 11**–**12)**
Heart rate [bpm]	228 ± 6	205 ± 6 **	212 ± 4 *
LVEDP [mmHg]	7 ± 1	10 ± 1	15 ± 1 ***^;##^
LVESP [mmHg]	112 ± 5	163 ± 4 ***	176 ± 8 ***
LVEDV [µL]	386 ± 21	526 ± 15 ***	552 ± 24 ***
LVESV [µL]	144 ± 12	190 ± 14 *	236 ± 29 ***^;##^
SW [mmHg × µL]	29,590 ± 2194	59,273 ± 2516 ***	62,686 ± 4024 ***
dP/dt max [mmHg/s]	7123 ± 210	10,388 ± 155 ***	10,630 ± 283 ***
dP/dt min [mmHg/s]	−6817 ± 183	−8537 ± 163 ***	−8533 ± 229 ***
dV/dt max [µL/s]	6864 ± 478	7541 ± 713	8097 ± 610
dV/dt min [µL/s]	−5279 ± 348	−6177 ± 273	−6066 ± 446
Tau [ms]	18 ± 0.5	18 ± 0.5	18 ± 0.5
slope LV-Ees [mmHg/µL]	0.21 ± 0.02	0.3 ± 0.03 *	0.19 ± 0.01 ^##^
LV-stiffness constant β_w_	0.38 ± 0.05	0.58 ± 0.05	0.64 ± 0.09 *
MAP [mmHg]	94 ± 5	133 ± 3 ***	139 ± 5 ***

A: late mitral inflow, bpm: beats per minute, dP/dt max: maximum derivative of change in systolic pressure over time, dP/dt min: minimum derivative of change in diastolic pressure over time, dV/dt max: maximum derivative of change in volume over time, dV/dt min: minimum derivative of change in volume over time, E: early mitral inflow, é: tissue Doppler mitral annulus velocity in early diastole, FAC: fractional area change, LV: left ventricle, LVEDP: left ventricular end-diastolic pressure, LVEDV: left ventricular end-diastolic volume, LVEF: left ventricular ejection fraction, LVESP: left ventricular end-systolic pressure, LVESV: left ventricular end-systolic volume, LVFS: left ventricular fractional shortening, LVID;d: end-diastolic left ventricular inner diameter, LVPW;d: end-diastolic left ventricular posterior wall, LVSV: left ventricular stroke volume, MAP: mean arterial pressure, slope LV-Ees: slope of left ventricular end-systolic elastance, SW: stroke work, Tau: time constant of left ventricular relaxation, TL: tibia length. All data are displayed as mean ± SEM; con denotes the lean control group; HFpEF denotes the obese control group; and HFpEF/Ela denotes the obese treatment group. * *p* < 0.05, ** *p* < 0.01, *** *p* < 0.001 vs. con, ^#^ *p* < 0.05, ^##^ *p* < 0.01 vs. HFpEF.

**Table 2:** Carotid morphology and relative mRNA expression of fibrosis associated genes.

**Carotid morphology**			
	**con**	**HFpEF**	**HFpEF/Ela**
Media thickness [µm]	53.27 ± 1.79	54.86 ± 2.86	56.33 ± 2.65
Internal circumference [mm]	2.16 ± 0.05	2.22 ± 0.08	2.30 ± 0.11
Number of laminae	4.85 ± 0.08	4.82 ± 0.10	4.86 ± 0.11
Thickness between laminae [µm]	10.97 ± 0.26	11.35 ± 0.54	11.59 ± 0.43
**Relative gene expression**			
	**con**	**HFpEF**	**HFpEF/Ela**
Col1a1	1.00 ± 0.13	1.21 ± 0.16	0.99 ± 0.13
Col1a2	1.00 ± 0.09	1.14 ± 0.10	1.04 ± 0.11
Col3a1	1.00 ± 0.10	1.28 ± 0.14	0.85 ± 0.09
Eln	1.00 ± 0.09	0.98 ± 0.07	1.02 ± 0.08
Fbn1	1.00 ± 0.11	0.93 ± 0.07	0.89 ± 0.05
Lox	1.00 ± 0.09	0.87 ± 0.07	1.12 ± 0.06 ^#^

Col1a1: collagen type I alpha 1; Col1a2: collagen type I alpha 2; Col3a1: collagen type III alpha 1; Eln: Elastin; Fbn1: Fibrillin 1, Lox: Lysyl oxidase. All data are displayed as mean ± SEM and expressed in arbitrary units (AU), with values for the con group normalized to 1; con denotes the lean control group; HFpEF denotes the obese control group; and HFpEF/Ela denotes the obese treatment group. ^#^ *p* < 0.05 vs. HFpEF.

## Data Availability

The original contributions presented in this study are included in the article. Further inquiries can be directed to the corresponding author.

## Abbreviations

The following abbreviations are used in this manuscript:

CO	Cardiac output
con	Control
E/é	Early mitral inflow velocity/early diastolic mitral annular velocity
Ela	Elamipretide
FAC	Fractional area change
GLP-1 RA	Glucagon-like peptide-1 receptor agonist
HFpEF	Heart failure with preserved ejection fraction
HFrEF	Heart failure with reduced ejection fraction
LV	Left ventricle
LVEDP	Left ventricular end-diastolic pressure
LVEDV	Left ventricular end-diastolic volume
LVEF	Left ventricular ejection fraction
LVESP	Left ventricular end-diastolic pressure
LVFS	Left ventricular fractional shortening
LVID;d	End-diastolic left ventricular inner diameter
LVPW;d	End-diastolic left ventricular posterior wall
LVSV	Left ventricular stroke volume
MAP	Mean arterial pressure
SGLT2i	Sodium–glucose cotransporter 2 inhibitor
slope LV-Ees	Slope of left ventricular end-systolic elastance
SW	Stroke work
Tau	Time constant of left ventricular relaxation
TL	Tibia length
ZSF1	Zucker fatty/spontaneously hypertensive heart failure F1 hybrid

## Appendix A

### Appendix A.1

**Table A1:** Animal characteristics at 20 weeks of age, prior to beginning of experiment.

Parameter	con (*n* = 10)	HFpEF (*n* = 12)	HFpEF/Ela (*n* = 12)
Body weight [g]	245 ± 3	425 ± 8 ***	434 ± 6 ***
LVEF [%]	75.8 ± 2	80.1 ± 2	81.3 ± 2
FAC [%]	69.9 ± 2	72.3 ± 3	74.3 ± 2
E/é	18.5 ± 1	26.3 ± 2 **	28.8 ± 2 ***

E: early mitral inflow, é: tissue Doppler mitral annulus velocity in early diastole, FAC: fractional area change, LVEF: left ventricular ejection fraction. All data are displayed as mean ± SEM; con denotes the lean control group; HFpEF denotes the obese control group; and HFpEF/Ela denotes the obese treatment group. ** *p* < 0.01, *** *p* < 0.001 vs. con.

### Appendix A.2

**Table A2:** Primer sequences used for real-time RT-PCR.

Gene	Sequence (5′-3′) Sense	Sequence (5′-3′) Antisense	Gene Bank Acc.-No.
*Cd68*	CACCAGTCATGGGAATGC	GGAATGAGAGAGCCAAGTG	NM_001031638
*Col1a1*	CTGCACGAGTCACACCGGAA	CCAATGTCCAAGGGAGCCAC	NM_053304
*Col1a2*	GTGGCAGCCAGTTTGAATAC	TGTTCTGAGAAGCACGGTTG	NM_053356
*Col3a1*	TGGCTGCACTAAACACACTG	CCAATGTCATAGGGTGCGAT	NM_032085
*Eln*	TCCCGGTGGAGTTGGTGTTG	CCCTGCTCCTCCAAGATCAC	NM_012722
*Fbn1*	CATGACCAACGGAGCAGATATTG	TCCCTGTTATGTCCGGAGTG	NM_031825
*Lox*	CGGTTACTTCCAGTACGGTCTC	CCGCCCTATATGCTGAACTG	NM_017061
*Myh6*	AGGACCAGGCCAATGAATAC	AGCTCGCCGTTCTCTGTCTG	NM_017239
*Myh7*	TGAATGAACACCGGAGCAAGG	GTCTGGACAGCTCCCCATTC	NM_017240
*Polr2a*	GGTATTGAGCAGATCAGCAAGG	CAATGCCCAGTACCGTGAAG	NM_001427041
*Sox9*	AGACCTCTGGGCAAGCTCTGG	CCCATTCTTCACCGACTTCC	NM_080403
*Tbp*	ATCACTCCTGCCACACCAGC	CGCAGTTGTTCGTGGCTCTC	NM_001004198
